# From Counting Dollars to Counting Sheep: Exploring Simultaneous Change in Economic Well-Being and Sleep among African American Adolescents

**DOI:** 10.1007/s40615-024-02212-9

**Published:** 2024-10-22

**Authors:** Morgan J. Thompson, Leanna M. McWood, Joseph A. Buckhalt, Mona El-Sheikh

**Affiliations:** 1 Department of Human Development & Family Science, Auburn University, 203 Spidle Hall, Auburn, AL 36849-5214 USA; 2 North Dakota State University, Fargo, ND 58105 USA

**Keywords:** Sleep Duration, Sleep Quality, Actigraphy, Socioeconomic Status, Black Youth, Adolescents

## Abstract

In the U.S.A., Black/African American adolescents disproportionately experience short and poor-quality sleep, and there is little understanding of why some experience decrements or improvements in sleep over time. Toward conducting culturally specific research and identifying processes that uniquely explain variability *within* a racial/ethnic group, we utilized a within-group design to examine socioeconomic status (SES) as a predictor of Black adolescents’ sleep. Few studies have examined change-on-change processes in sleep over time. Contributing to the literature in novel ways, we assessed the predictive effect of change in SES over one year on changes in four actigraphy-derived sleep parameters over the same time period. Participants were 218 Black adolescents (Time 1: *M*_*age*_ = 17.09 years; 54.6% female) and their mothers from socioeconomically diverse backgrounds. Adolescents participated in two-waves of data collection spaced approximately one year apart. At each wave, we assessed economic well-being (used to index SES) and sleep using 7 nights of actigraphy from which we derived measures of sleep duration (minutes) and quality (efficiency, long-wake episodes, activity). Latent difference score analyses revealed that adolescents experiencing increases in SES over one year exhibited decreases in both long-wake episodes and sleep activity over one year. Findings suggest that individual differences in change in SES explain individual differences in change in adolescents’ sleep quality. Notably, findings highlight the utility of within-group designs for identifying culturally specific processes that predict improvements in sleep quality in a sample at disproportionate risk for sleep and health disparities.

**Supplementary Information:**

The online version contains supplementary material available at 10.1007/s40615-024-02212-9.

 Sleep problems (i.e., short, low-quality sleep) among adolescents are pervasive [1, 2]. Considerable evidence suggests that socioeconomically disadvantaged and minoritized groups may disproportionately experience poor sleep [3–8], and there is a critical need to identify factors that may ameliorate racial/ethnic (hereafter referred to as race/racial) disparities in health [9]. Studies testing these associations have primarily emphasized between-group designs comparing differences across racial groups [5, 10] and objective assessments of socioeconomic status (SES; i.e., family income, parental education) [4, 6, 11]. Toward conducting culturally specific research, utilizing within-group designs will be critical for identifying processes that uniquely explain variability *within* racial groups [5, 10, 12, 13]. There is considerable variability in sleep among Black/African American (hereafter referred to as Black) youth, and it will be important to pinpoint variables that predict better and worse sleep for Black youth over time [5, 10]. Although Black families persistently experience greater socioeconomic risk [13, 14], subjective assessments of SES may provide important insights into the heterogeneity of Black families’ unique economic experiences [11, 15]. Given SES is associated with both race and sleep disparities, examining individual differences in change in SES may be one promising predictor of individual differences in change in sleep among Black youth.

Studies examining how changes in individual differences in variables (including SES) predict changes in sleep parameters over time are scarce [16]. Addressing the need for a more comprehensive understanding of factors that predict change in adolescents’ sleep over time, we utilized a within-group design to examine how change-on-change processes between SES and sleep uniquely unfold within a minoritized sample. Specifically, we tested individual differences in change in subjective SES (i.e., maternal perceived economic well-being) over one year as a predictor of individual differences in change in four sleep parameters over the same time period in a sample of Black adolescents. We examined actigraphy-derived sleep duration (*minutes*—total number of minutes scored as sleep between sleep onset and wake time) and quality (*efficiency*—percentage of time between sleep onset and wake time scored as sleep, *long-wake episodes*—number of wake episodes ≥ 5 min, *activity*—percentage of sleep epochs with activity). Perceived economic well-being is a multidimensional construct reflecting financial setbacks, adjustments, and strain [10]. Specifically, we measured perceived economic well-being as families’ difficulty making ends meet, ability to meet material needs, and financial cutbacks [17].

SES is a multifaceted construct comprising monetary resources, social prestige, perceptions of economic well-being, educational status, and broader community variables (e.g., crime rates). Although most research examining associations between SES and sleep has examined monetary resources and education [4, 11], subjective assessments provide important insights for understanding the effect of economic pressure and stress on families [11, 15]. For instance, families may perceive considerable financial strain even in the absence of income-based poverty [18] or following brief spells of income-based poverty [19]. Moreover, according to the family stress model, parental perceived economic hardship is proposed to disrupt youth development by increasing household stress levels and family challenges (e.g., parental mental health, interparental conflict, harsh parenting) [20]. Thus, subjective economic adversity is a significant source of stress that can have a pivotal impact on youths’ sleep. On the other hand, economic well-being may operate as a protective factor that offsets the risk of poor sleep problems [20]. Indeed, many studies indicate that perceived economic well-being is associated with better sleep, or similarly, perceived financial strain predicts less optimal sleep [6, 22–25].

Most research that has examined sleep in minoritized youth has utilized a between-group design that uses race as a grouping variable to identify differences from a majority group membership [5, 10]. Specifically, all individuals within a racial group are viewed as equally vulnerable, overlooking within-group heterogeneity resulting from sociohistorical and culturally relevant factors [12, 13, 26, 27]. In comparison, within-group analyses may offer a powerful tool for conducting culturally specific research. This approach emphasizes identifying processes and variables such as SES that predict within-group variations [5, 10, 12, 13, 28]. For instance, exploring the variability in economic experiences and pressures among Black families could provide important insights into individual differences in Black youths’ sleep and explain why some sleep better than others. Nevertheless, studies have yet to test how within-group variations in predictive processes like SES may inform within-group variations in sleep. Accordingly, there remains a critical need to catalog variability in stress exposure and the heterogeneity in health disparities among minoritized samples [12, 26, 29, 30].

Contributing to this small literature, we examined within-group variations in change in parental perceived economic well-being (hereafter referred to as SES) over one year as a predictor of within-group variations in change in four actigraphy-derived sleep parameters over the same time period in a large sample of Black adolescents. This study has several novel features. First, we utilized a within-group analysis design to examine relations between SES and sleep in a historically minoritized sample at greater risk for experiencing structural barriers [30] known to affect sleep [31]. Second, studies have primarily utilized residualized change models to determine how prior levels of SES predict stability over time in sleep. We utilized latent difference score modeling to examine how individual differences in change in SES are associated with individual differences in change in sleep [32, 33]. Finally, by focusing on within-group analyses and individual differences in change, we move away from a deficit-oriented explanation for sleep disparities that assumes all minoritized youth are at-risk and rather take a closer look at within-group variability to identify sociocultural processes (e.g., SES) that increase or decrease risk and offer protection [13]. We hypothesized that increases in SES over one year would be associated with improvements in sleep duration (minutes) and quality (efficiency, long-wake episodes, activity) over time. Conversely, we expected decreases in SES would predict worsening sleep.

## Method

### Participants

Data for this study were drawn from two independent longitudinal investigations. Most study procedures were similar across the two samples. Participants were recruited by sending letters home with youth attending local elementary schools. Families were not eligible to participate if children were diagnosed with a chronic illness, learning disability, or sleep disorder as reported by mothers at recruitment. All study procedures were approved by the university’s Institutional Review Board. Reasons for attrition were similar across studies such as scheduling conflicts, inability to locate people, moving, or lack of interest. Each study is described in further detail below.

#### Study 1

The Auburn University Sleep Study aims to examine health disparities in sleep from middle childhood to early adulthood across five time points [34]. For the first wave (W1) of the study (2009–10 school year), 282 Black and White participants were recruited. At W4, an additional 126 families were recruited from the same school districts using the same methods and eligibility criteria to increase power. The fourth and fifth waves of the study, relevant to the present paper, were collected in 2017–18 and 2019–20 respectively. The overall sample was composed of 323 participants at W4 (i.e., T1 of the present study; *M*_*age*_ = 17.38 years, *SD* = 10.18 months; 85.8% attending secondary school) and W5 (i.e., T2 of the present study; *M*_*age*_ = 18.69 years, *SD* = 11.88 months; 63.3% attending secondary school). There was approximately a 1-year interval between T1 and T2 (*M* = 1.29 years, *SD* = 121 days). The sample was comprised of 41.5% Black and 58.5% White.

#### Study 2

The Family Stress and Youth Development Study examines family stress and youth development from middle childhood through early adulthood across eight time points [35]. For the first wave of the study, 251 Black and White participants were recruited in 2005. Eligibility criteria included residing in two-parent homes for ≥ two years. To increase sample size at W4, an additional 53 families were recruited using the same recruitment procedures and eligibility criteria as the initial W1 sample. The newly recruited participants matched the original sample demographics on race and age. The fifth and sixth waves of data, relevant to the present paper, were collected in 2013–14 and 2014–15. The sample was composed of 313 possible participants at W5 (i.e., T1 of the present study; *M*_*age*_ = 16.24 years, *SD* = 9.72 months; 98.4% attending secondary school) and W6 (i.e., T2 of the present study; *M*_*age*_ = 17.19 years, *SD* = 9.6 months; 57.5% attending secondary school).^1^ There was approximately a 1-year interval between T1 and T2 (*M* = 0.97 years, *SD* = 20 days). The sample was comprised of 33.2% Black and 66.8% White.

For the present study, due to similarities across sample characteristics and study design, we combined the two datasets to create a larger, more powerful sample size for testing primary study hypotheses. The current analysis utilized a subset of participants that only included data from Black participants. This resulted in an analytic sample of 218 participants (39% derived from Study 1) (*M*_*age*_ = 17.09 years, *SD* = 11.03 months; 54.6% female, 45.4% male). Hereafter, all information provided detail only the current analytic sample. The income-to-needs ratio considers the family income in relation to the number of individuals residing in the family (U.S. Department of Commerce, 2012). The income-to-needs ratio in the sample included 33% living in poverty, 26.3% low income, 34.1% middle class, and 6.6% middle-upper class or higher.

Adolescents who participated at T1 but did not participate at T2 reported greater T1 financial strain (*t*(58.11) = − 1.94, *p* = .03, *M*_*Missing*_ = − 0.48, *M*_*Not missing*_ = − 0.13), and equal variances could not be assumed across groups according to the Levene’s Test (F = 8.68, *p* = .004). Adolescents did not differ on any other study variables at T1 including all sleep parameters and all covariates. Females obtained more sleep minutes at T1 (*t*(185) = 2.80, *p* = .003, *M*_*Females*_ = 394.23, *M*_*Males*_ = 370.80) and T2 (*t*(185) = 2.80, *p* = .003, *M*_*Females*_ = 387.47, *M*_*Males*_ = 366.03). Males and females did not differ on any additional sleep parameters, SES, or covariates. There were few differences between Study 1 and Study 2. Participants in Study 1 reported higher T2 sleep efficiency (*t*(152) = 2.51, *p* = .007, *M*_*Study1*_ = 92.11, *M*_*Study2*_ = 89.06) and fewer T2 long-wake episodes (*t*(89.57) = − 1.89, *p* = .031, *M*_*Study1*_ = 2.09, *M*_*Study2*_ = 2.68).^2^

### Procedures

Data for both time points in both studies were collected during the school year excluding holidays and school breaks. We received parental consent and adolescent assent. Participants were compensated monetarily for their participation. In both studies adolescents were mailed actigraphs that they were instructed to wear for seven consecutive nights on their non-dominant wrist. Adolescents completed a sleep diary each morning, which was used to corroborate sleep onset and wake time. Each day’s medication was used for acute illnesses was also reported in the sleep diary. Soon after completing actigraph assessments, adolescents and their mothers visited our on-campus laboratory where they participated in lab tasks and completed surveys. Adolescents and their mothers also had the option to respond to surveys remotely. For brevity, only relevant study procedures are described.

### Measures

#### SES (Perceived Economic Well-being)

To assess SES, we utilized three established scales: Can’t Make Ends Meet, Material Needs, and Financial Cutbacks [17]. The Can’t Make Ends Meet subscale measured the difficulty the family had paying for financial necessities such as bills in the past year. The subscale included 3 items: (1) “Our income never seems to catch up with our expenses” rated on a 5-point scale (1 = *strongly agree* to 5 = *strongly disagree*), (2) “Think back over the past year and tell us how much difficulty you had with paying your bills” rated on a 5-point scale (1 = *a great deal of difficulty* to 5 = *no difficulty at all*), (3) “Think again over the past 12 months. Generally, at the end of each month do you end up with.” with response options ranging from 1 (*not enough to make ends meet*) to 4 (*more than enough to make ends meet*). Items were standardized and averaged (*α*s = 0.78 and 0.80 at T1 and T2, respectively). The Material Needs subscale examined the family’s overall economic situation (7 items; e.g., “We have enough money to afford the kind of car we need”, “My family has enough money to afford the kind of home we would like to have”, “We have enough money to afford the kind of food we should have”). Items were rated on a 5-point scale (1 = *strongly agree* to 5 = *strongly disagree*) and were each reverse scored then standardized and averaged (*α*s = 0.92 and 0.93 at T1 and T2, respectively). The Financial Cutbacks subscale assessed changes the family might have made in the past year because of their financial situation (22 items; e.g., “Reduced or eliminated medical insurance”, “Used savings to meet daily living expenses”, “Sold possessions or cashed in life insurance”, “Changed food shopping or eating habits to save money”). Mothers reported on financial cutbacks through agreement (1 = *yes*, 2 = *no*) with each item. The three scales were moderately correlated with one another (*r*s = 0.35 to 0.64). Scores were averaged (*α*s = 0.83 at T1 and T2), and higher scores reflected fewer cutbacks made in the past year (i.e., greater economic wellbeing) whereas lower scores reflected perceived financial strain. Previous research utilizing this scale demonstrates both reliability and validity [17, 36]. Consistent with established practice [37], the subscales were standardized and the three scales were averaged to create an overall perceived economic well-being score (*α*s = 0.89 and 0.91 at T1 and T2, respectively).

#### Sleep

Motionlogger Octagonal Basic actigraphs (Ambulatory Monitoring Inc., Ardsley, NY) were utilized to assess sleep, which measured movement over 1-minute epochs using zero-crossing mode and Sadeh’s scoring algorithm [38] in ACTme software (Action, W2, 2002). An experienced coder used movement detected by the actigraph to manually set sleep onset (i.e., the first of three consecutive minutes scored as sleep) and wake time (the last minute of the last five consecutive minutes scored as sleep). The sleep diary was also utilized to corroborate sleep times—lack of corroboration (> 30 min difference between onset or wake time in the sleep diary and actigraphy) was uncommon (average of 13 adolescents per night). Sleep data were included for participants if they had ≥ three nights of valid actigraphy data; otherwise, they were treated as missing. At T1, adolescents had on average 5.13 (*SD* = 2.00) nights of actigraphy data. 32% of youth had the mode of 7 nights of data, and sleep data were treated as missing for 9% of participants. Missing data were due to forgetting to wear the device or actigraph malfunctions (the latter was rare). At T2, adolescents had on average 4.64 (*SD* = 2.08) nights of actigraphy data. 25% of youth had the mode of 6 nights of data, and sleep data were treated as missing for 15% of participants.

Based on the software and accompanying manual, we derived four sleep variables. *Sleep minutes* was calculated as the number of minutes scored as sleep from sleep onset to wake time. *Sleep efficiency* was measured as the percentage of minutes spent asleep between sleep onset and wake time. *Long-wake episodes* reflected the number of wakefulness periods lasting ≥ 5 min. *Sleep activity* assessed the percentage of epochs (i.e., minutes) with activity. All sleep variables were averaged across available nights.

#### Covariates

Covariates in the present study included mother report of youth sex assigned at birth (0 = female, 1 = male), study (0 = Study 1, 1 = Study 2), and standardized body mass index (*z*BMI).

### Statistical Analysis

Primary analyses were conducted using structural equation modeling (SEM) in Mplus Version 8.4 [39]. We used full information maximum likelihood (FIML) estimation to handle missing data and retain all 218 participants for analyses. Data in our sample were missing for 18% of values, which is within the acceptable range for use of FIML [40]. Data were missing completely at random, based on Little’s MCAR test, *χ2*(121) = 132.24, *p* = .23 [41, 42]. Study variables were assessed for normality and all variables fell within an acceptable range (i.e., skewness value < 2.0).

**Fig. 1:**
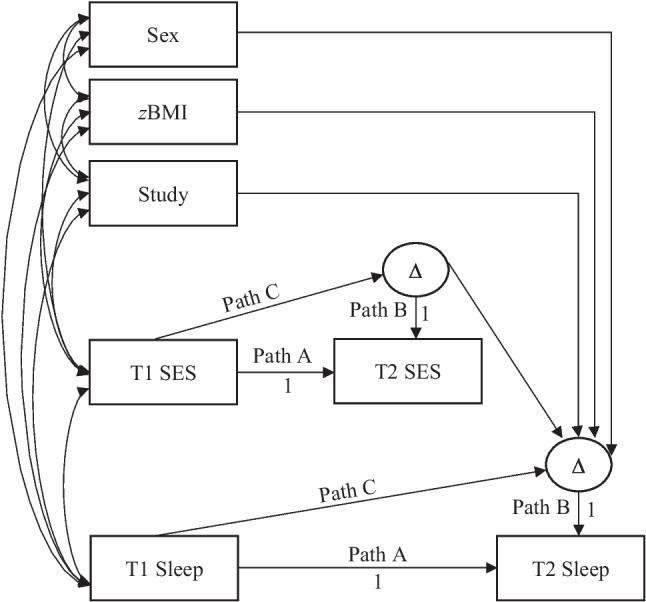
Depiction of structural equation model using latent difference score analyses to estimate the predictive effect of change in socioeconomic status (SES) from Time 1 (T1) to Time 2 (T2) on change in sleep from T1 to T2; Path A depicts the autoregressive path between T1 and T2; Path B depicts the structural path between the latent change score and the T2 assessment; Path C depicts the proportional change path between the T1 assessment and the latent change score; *z*BMI = standardized body mass index; ∆ = latent change

To test the predictive effect of SES on adolescents’ sleep, we used latent difference score (LDS) and followed well-established procedures [32, 33]. LDS analyses capture change in variables across two measurement occasions and provide assessments of (a) the average change in the overall sample, and (b) the degree of variability in change across the sample (specifically, individual differences in intraindividual change). We estimated LDS change analyses from T1 to T2 for SES, sleep minutes, sleep efficiency, long-wake episodes, and sleep activity. Following established guidelines [33], we regressed the T2 assessment of each variable onto the T1 assessment of the same variable (Path A, Fig. 1) and its latent change score (Path B, Fig. 1). Both paths were constrained to 1. To estimate proportional change, we specified a structural path between the T1 assessment of each variable and its latent change score (Path C, Fig. 1). To test our primary aim of examining the predictive effect of SES, we estimated regression paths between LDS change in SES and LDS change in each sleep variable. We estimated sex assigned at birth, *z*BMI, and study project as predictors of LDS change in sleep variables. We estimated correlations among all exogenous variables. For a depiction of the analytic model, see Fig. 1. Sleep variables were assessed individually in four consecutive models to facilitate the interpretation of the main effect of SES.^3^

## Results

### Descriptive Analyses

Table 1 provides the means, standard deviations, and correlations for study variables. Stability over time ranged from modest to moderate for SES and all sleep variables. Correlations among sleep variables were mostly significant.

**Table 1:** Correlations and descriptive statistics for study variables

	M	SD	1	2	3	4	5	6	7	8	9	10	11	12
1. Youth Sex	0.50	0.50	–											
2. Standardized Body Mass Index	0.49	0.49	–0.10	–										
3. Study	1.12	1.12	–0.05	0.02	–									
4. Socioeconomic Status T1	1.24	1.24	–0.01	–0.20^*^	0.07	–								
5. Socioeconomic Status T2	1.31	1.31	–0.10	–0.12	0.03	0.62^*^	–							
6. Sleep Minutes T1	58.01	58.01	–0.20^*^	–0.02	–0.03	0.16^*^	0.13	–						
7. Sleep Minutes T2	58.48	58.48	–0.18^*^	–0.08	–0.12	–0.07	–0.04	0.42^*^	–					
8. Sleep Efficiency T1	7.25	7.25	–0.08	–0.04	–0.04	0.14^†^	0.10	0.55^*^	0.22^*^	–				
9. Sleep Efficiency T2	7.50	7.50	–0.11	–0.05	–0.20^*^	–0.05	0.08	0.20^*^	0.53^*^	0.29^*^	–			
10. Long-Wake Episodes T1	2.28	2.28	0.03	0.05	0.01	–0.13^†^	–0.10	–0.42^*^	–0.23^*^	–0.89^*^	–0.35^*^	–		
11. Long-Wake Episodes T2	2.33	2.33	0.07	0.06	0.17^*^	0.05	–0.10	–0.11	–0.40^*^	–0.28^*^	–0.92^*^	0.37^*^	–	
12. Sleep Activity T1	33.52	33.52	0.01	–0.01	–0.04	–0.13	–0.15^†^	–0.29^*^	–0.13	–0.67^*^	–0.19^*^	0.62^*^	0.21^*^	–
13. Sleep Activity T2	34.69	34.69	0.05	–0.04	–0.05	0.09	–0.11	–0.01	–0.33^*^	–0.10	–0.69^*^	0.12	0.67^*^	0.44^*^

Adolescent sex coded as 0 = female, 1 = male; study coded as 0 = Auburn University Sleep Study, 1 = Family Stress and Youth Development Study. ^†^ = *p* < .10. ^*^ = *p* < .05

### Preliminary Analyses: Latent Change in Socioeconomic Status and Sleep

To characterize the average sample change and variability (or individual differences) in change, we first tested univariate unconditional LDS models for SES and the four sleep variables (see Table 2). Each of the five models were fully saturated and thus fit statistics are not reported. Mothers on average reported nonsignificant increases in SES over one year. Over one year, adolescents on average exhibited nonsignificant decreases in sleep minutes and nonsignificant increases in sleep efficiency, long-wake episodes, and sleep activity. Despite nonsignificant average change in each variable, there was significant variability in LDS change in SES,^4^ sleep minutes, sleep efficiency, long-wake episodes, and sleep activity. Significant variability (or individual differences) in LDS change indicates considerable deviation around mean levels (i.e., youth exhibit different rates of change over time), which is necessary for using change scores as a predictor or outcome [43, 44].

**Table 2:** Unstandardized parameter estimates for latent difference scores

Variables	Average Change (µ)	Variability in Change (σ^2^)
Socioeconomic Status	0.02	1.24^***^
Sleep Minutes	–4.58	3879.64^***^
Sleep Efficiency	0.33	72.46^***^
Long-Wake Episodes	0.04	3.43^***^
Sleep Activity	1.05	129.48^***^

^***^ = *p* < .001

### Primary Analyses: Change in Socioeconomic Status as a Predictor of Change in Sleep

Given the documentation of significant differences in intraindividual change in SES and each sleep outcome, we proceeded to test the predictive effect of change in SES from T1 to T2 on change in each of the sleep variables from T1 to T2 using LDS modeling (see Fig. 1). Results from latent difference score analyses are provided in Table 3.^5^ Each model provided a good fit to the data. Increases in SES predicted decreases in adolescents’ long-wake episodes (*ß* = –0.17, *p* = .02) and sleep activity (*ß* = –0.19, *p* = .01). There was a near significant effect between increases in SES and increases in sleep efficiency (*ß* = 0.11, *p* = .09). Change in SES failed to predict subsequent change in sleep minutes.

**Table 3:** Latent difference score analyses examining sleep outcomes individually

	∆Sleep Minutes		∆Sleep Efficiency		∆Long-Wake Episodes		∆Sleep Activity	
	*ß*	*SE*	*ß*	*SE*	*ß*	*SE*	*ß*	*SE*
Proportional Change	–0.56^***^	0.07	–0.58^***^	0.09	–0.53^***^	0.10	–0.50^***^	0.07
Youth Sex	–0.10	0.07	–0.06	0.07	0.03	0.07	–0.01	0.07
Standardized Body Mass Index	–0.10	0.07	–0.05	0.08	0.06	0.09	–0.00	0.07
Study	–0.05	0.07	–0.14^*^	0.07	0.12	0.08	–0.06	0.07
∆ Socioeconomic Status	0.07	0.08	0.11^†^	0.07	–0.17^*^	0.07	–0.19^**^	0.07
Fit Indices								
χ^2^	5.81		3.94		4.01		5.34	
*df*	5		5		5		5	
χ^2^ /*df*	1.16		0.79		0.80		1.07	
RMSEA	0.03		0.00		0.00		0.02	
CFI	0.99		1.00		1.00		0.99	

RMSEA = root mean square error of approximation; CFI = comparative fit index. ^†^ = *p* < .10. ^*^ = *p* < .05. ^**^ = *p* ≤ .01. ^***^ = *p* < .001

## Discussion

Research indicates considerable differences in sleep-wake problems across racial groups [4, 5, 8]. However, most work has emphasized between-group approaches that utilize race as a grouping variable and assume individuals within racial groups are equally vulnerable [5, 13]. These designs do not consider sociohistorical and cultural contexts that may account for variability within a minoritized sample at greater risk for socioeconomic disadvantage and sleep disparities [5, 10, 12, 13, 28]. Consequently, there is little understanding about what variables predict better and worse sleep for Black youth longitudinally [5, 10]. Research has also primarily examined objective assessments of SES and utilized analytic models that focus on stability over time in sleep—the assessment of change-on-change processes in sleep is scarce yet is key to identifying variables and mechanisms of vulnerability and protection [16]. Focusing on a sample of Black adolescents, we utilized LDS modeling and a within-group design to examine individual differences in SES as a predictor of individual differences in adolescents’ sleep. Novel findings indicate that change in SES over one year was associated with simultaneous change in sleep quality (long-wake episodes and sleep activity) over the same time period.

Stress process models suggest that exposure to stress is not uniform across disadvantaged groups and this variability may lend a hand to predicting differential vulnerability and protection [29]. Although national data suggests that Black families persistently experience greater socioeconomic risk [13, 14], findings from the current study suggest that Black adolescents in homes experiencing increases in financial strain were most at risk for decreases in sleep quality. These youth likely have access to fewer protective psychosocial and material resources that may reduce some of the pervasive institutional and structural barriers [29]. Findings from the current study are consistent with the family stress model [20] and prior research [22, 24], and indicate that the effects of economic hardship trickle down to disrupt adolescent development—specifically their sleep quality in our investigation. Conversely, and importantly, our findings may also be interpreted from a protective lens whereby increases in SES were associated with improvements in sleep underscoring the role SES may play in mitigating sleep problems among Black adolescents and the critical need to identify additional variables that may reduce racial disparities in health [9].

While some have found that SES is also associated with sleep minutes [23, 24], we did not. One possible explanation for this discrepancy is the within-group design of the current investigation. Within-group designs emphasize factors that may account for variability in sleep outcomes within racial groups; as a result, findings may diverge from those conducted using between-group designs [45]. Second, adolescents may compensate for a poor night’s sleep (periods of wakefulness and/or high activity) by sleeping longer [46], although this was not tested in the current study. As such, it is possible that stress associated with socioeconomic disadvantage may have a stronger observable effect on nightly sleep disruption than actual time or percentage of the sleep period spent asleep. Clearly, these plausible explanations are tentative pending replication.

Study findings should be considered within the context of the sample and methodology. First, we examined associations between SES and adolescents’ sleep in a sample of Black youth residing in small towns and semi-rural areas in the southeastern U.S.A, which has a unique history of minoritization raising questions about generalizability to other groups. Second, analyses were correlational in nature and future research may gain further insights from experimental designs. Third, future research should consider individual differences in other indices of SES such as neighborhood and wider community factors, which have been associated with sleep [3, 7, 47]. Fourth, we measured individual differences in SES and sleep simultaneously, and it will be important for future research to explore predictors of change in sleep during subsequent developmental periods. Finally, it is essential for future research to consider other institutional and structural barriers (e.g., labor market, regional culture, observed neighborhood characteristics, anticipated prejudice) that are likely associated with sleep and health disparities among Black youth [47–49]. Despite these limitations, there are several strengths to our study. Utilization of a within-group approach furthered an understanding of how SES may function to improve sleep outcomes specifically among Black adolescents. Additionally, findings remained generally consistent across a range of sensitivity analyses including additional controls for age, school status, and income-to-needs ratio. Lastly, we assessed multiple indices of actigraphy-derived sleep, which strengthens conclusions.

A significant contribution of this paper is the finding that adolescents from families that had decreases in SES may be at heightened risk for poor sleep quality. Nevertheless, the other side of the coin suggests that increases in SES may protect youth from increasing sleep problems in a historically minoritized sample. While many institutional and structural barriers may be related to increased risk for poor sleep, this study highlights the predictive role the family’s financial situation may play in offsetting this risk and improving sleep among Black adolescents. These findings underscore the need for intervention and prevention efforts to rectify institutional and structural barriers (e.g., food deserts, school quality, neighborhood safety) [50] and associated socioeconomic disadvantage, which in turn could ameliorate risk for sleep problems in minoritized families at disproportionate risk for health disparities.

## Electronic Supplementary Material

Below is the link to the electronic supplementary material.

Supplementary Material 1

Supplementary Material 2

Supplementary Material 3

Supplementary Material 4

Supplementary Material 5

Supplementary Material 6

## Data Availability

Data are not available yet for sharing with others. Per National Institutes of Health data sharing guidelines, they will be available to other scholars at a later date after the completion of the ongoing longitudinal study.

## Footnotes

1 Given mean age and school status differences across the two studies, a set of sensitivity analyses were conducted to determine whether these variables substantively influenced primary study findings. Results from sensitivity analyses were generally consistent with findings reported in text (Online Resource 1).

2 We provide a side-by-side comparison of Study 1 and Study 2 characteristics for participants in the final analytic sample (Online Resource 2). We also conducted sensitivity analyses within each dataset to determine how study findings varied across samples. First, unstandardized parameter estimates for latent difference scores were generally comparable across Study 1 and Study 2 (Online Resource 3). A few differences emerged. We observed nonsignificant improvements in SES, sleep efficiency, and long-wake episodes among Study 1 participants, whereas Study 2 showed nonsignificant decrements in these variables. Additionally, we found greater variability in change over time for SES, sleep efficiency, long-wake episodes, and sleep activity in Study 1 and greater variability in sleep minutes in Study 2. Second, primary study findings in text were generally consistent when assessed within the Study 1 subset, but not within the Study 2 subset (Online Resource 4). Inconsistencies may in part be due to the considerably smaller sample size for Study 2, which would not only restrict variability (as shown in unstandardized latent change score estimates) but also reduce corresponding power to detect a significant effect. Relatedly, there was a wider SES range in Study 1, which may increase variability for correlational analyses.

3 Sensitivity analyses were conducted to determine whether primary study findings substantively differed if all objective sleep outcomes were assessed simultaneously. Results from sensitivity analyses were generally consistent with findings reported in text (Online Resource 5).

4 We additionally examined latent change in income-to-needs ratios for an objective assessment of family SES; however, there was neither significant change, on average, over the 1-year period (*µ* = 0.17, *p* = .22) nor significant individual differences in LDS change in income-to-needs (*σ*^*2*^ = 2.02, *p* = .09). Accordingly, we did not examine LDS change in income-to-needs ratios as a predictor of LDS change in sleep outcomes.

5 Sensitivity analyses examined whether primary study findings substantively differed when controlling for T1 income-to-needs ratio. Results from sensitivity analyses were generally consistent with findings reported in text (Online Resource 6).
