# Supplementary material for: From Counting Dollars to Counting Sheep: Exploring Simultaneous Change in Economic Well-Being and Sleep among African American Adolescents
Source: J Racial Ethn Health Disparities. 2024 Oct 22;12(6):4199–208. doi: 10.1007/s40615-024-02212-9 (PMC12644149; doi:10.1007/s40615-024-02212-9)
Supplement: Supplementary file 1 — Supplementary Material 1 [file 40615_2024_2212_MOESM1_ESM.docx]

| *Latent Difference Score Analyses Examining Sleep Outcomes Individually While Controlling for Age and School Status* | | | | | | | | |
| --- | --- | --- | --- | --- | --- | --- | --- | --- |
|  | ∆Sleep  Minutes | | ∆Sleep  Efficiency | | ∆Long-Wake  Episodes | | ∆Sleep  Activity | |
|  | *ß* | *SE* | *ß* | *SE* | *ß* | *SE* | *ß* | *SE* |
| Proportional Change | –.56^***^ | .07 | –.57^***^ | .09 | –.53^***^ | .10 | –.50^***^ | .06 |
| Sex | –.09 | .08 | –.04 | .07 | .02 | .07 | –.03 | .07 |
| Body Mass Index | –.10 | .07 | –.04 | .08 | .05 | .09 | –.01 | .06 |
| Study | –.06 | .08 | –.24^***^ | .07 | .20^*^ | .08 | .06 | .08 |
| T1 Age | .07 | .21 | .18 | .16 | –.20 | .17 | –.29^†^ | .17 |
| T2 Age | –.07 | .22 | –.29^†^ | .17 | .27 | .19 | .44^**^ | .17 |
| T1 School Status | .06 | .04 | .12^*^ | .06 | –.10^†^ | .05 | –.15^**^ | .05 |
| T2 School Status | –.06 | .08 | –.18^*^ | .09 | .15^†^ | .08 | .11 | .08 |
| ∆Perceived Economic Wellbeing | .06 | .08 | .10 | .07 | –.16^*^ | .07 | –.16^*^ | .07 |
|  |  |  |  |  |  |  |  |  |
| Fit Indices |  |  |  |  |  |  |  |  |
| χ^2^ | 15.13 |  | 13.84 |  | 13.26 |  | 17.02 |  |
| *df* | 11 |  | 11 |  | 11 |  | 11 |  |
| χ^2^ /*df* | 1.38 |  | 1.26 |  | 1.21 |  | 1.55 |  |
| RMSEA | .04 |  | .03 |  | .03 |  | .05 |  |
| CFI | .96 |  | .97 |  | .98 |  | .95 |  |
| *Note*. RMSEA = root mean square error of approximation; CFI = comparative fit index. ^†^ = *p* < .10. ^*^ = *p* ≤ .05. ^**^ = *p* < .01. ^***^ = *p* ≤ .001. | | | | | | | | |
