# Supplementary material for: From Counting Dollars to Counting Sheep: Exploring Simultaneous Change in Economic Well-Being and Sleep among African American Adolescents
Source: J Racial Ethn Health Disparities. 2024 Oct 22;12(6):4199–208. doi: 10.1007/s40615-024-02212-9 (PMC12644149; doi:10.1007/s40615-024-02212-9)
Supplement: Supplementary file 2 — Supplementary Material 2 [file 40615_2024_2212_MOESM2_ESM.docx]

| *Side-by-Side Comparison of Study 1 and Study 2 Characteristics for Final Analytic Sample* | | |
| --- | --- | --- |
|  | Study 1 (*N* = 133) | Study 2 (*N* = 85) |
|  | % or *M* (*SD*) | % or *M* (*SD*) |
| Demographics |  |  |
| T1 age (in years) | 17.30 (.95) | 16.72 (.75) |
| T2 age (in years) | 18.63 (1.08) | 17.61 (.72) |
| % Female | 53% | 58% |
| T1 standardized body mass index | .91 (1.21) | .95 (.95) |
| T1 income-to-needs ratio | 1.86 (1.33) | 1.92 (1.20) |
| T2 income-to-needs ratio | 2.13 (2.05) | 2.14 (1.22) |
| Primary Study Variables |  |  |
| T1 socioeconomic status | –.24 (1.30) | –.06 (1.13) |
| T2 socioeconomic status | –.19 (1.37) | –.11 (1.20) |
| T1 sleep minutes | 384.67 (55.46) | 381.43 (63.13) |
| T2 sleep minutes | 383.77 (59.68) | 369.56 (56.00) |
| T1 sleep efficiency | 90.95 (7.28) | 90.37 (7.24) |
| T2 sleep efficiency | 92.11 (6.31) | 89.06 (8.75) |
| T1 long-wake episodes | 2.27 (1.61) | 2.29 (1.99) |
| T2 long-wake episodes | 2.09 (1.34) | 2.69 (2.19) |
| T1 sleep activity | 33.80 (11.04) | 32.98 (10.52) |
| T2 sleep activity | 35.17 (10.56) | 33.96 (12.06) |
| Study 1: Auburn University Sleep Study. Study 2: Family Stress and Youth Development Study. | | |
