# Supplementary material for: From Counting Dollars to Counting Sheep: Exploring Simultaneous Change in Economic Well-Being and Sleep among African American Adolescents
Source: J Racial Ethn Health Disparities. 2024 Oct 22;12(6):4199–208. doi: 10.1007/s40615-024-02212-9 (PMC12644149; doi:10.1007/s40615-024-02212-9)
Supplement: Supplementary file 3 — Supplementary Material 3 [file 40615_2024_2212_MOESM3_ESM.docx]

| *Unstandardized Parameter Estimates for Latent Difference Scores for Study 1 and Study 2 Separately* | | | | | | |
| --- | --- | --- | --- | --- | --- | --- |
|  |  | Study 1 | |  | Study 2 | |
| Variables |  | Average Change (*µ*) | Variability in Change (*σ*^2^) |  | Average Change (*µ*) | Variability in  Change (*σ*^2^) |
| Socioeconomic Status |  | .11 | 1.37^***^ |  | –.19 | .89^**^^*^ |
| Sleep Minutes |  | –3.83 | 3596.48^***^ |  | –6.19 | 4426.98^***^ |
| Sleep Efficiency |  | 1.20 | 74.37^***^ |  | –.81 | 58.80^**^ |
| Long-Wake Episodes |  | –.18 | 3.44^***^ |  | .34 | 2.98^**^^*^ |
| Sleep Activity |  | 1.28 | 153.23^***^ |  | .77 | 78.49^***^ |
| *Note*. Study 1: Auburn University Sleep Study. Study 2: Family Stress and Youth Development Study.  ^**^ = *p* < .01. ^***^ = *p* < .001. | | | | | | |
