# Supplementary material for: From Counting Dollars to Counting Sheep: Exploring Simultaneous Change in Economic Well-Being and Sleep among African American Adolescents
Source: J Racial Ethn Health Disparities. 2024 Oct 22;12(6):4199–208. doi: 10.1007/s40615-024-02212-9 (PMC12644149; doi:10.1007/s40615-024-02212-9)
Supplement: Supplementary file 4 — Supplementary Material 4 [file 40615_2024_2212_MOESM4_ESM.docx]

| *Latent Difference Score Analyses Examining Sleep Outcomes* *for Study 1 and Study 2 Separately* | | | | | | | | |
| --- | --- | --- | --- | --- | --- | --- | --- | --- |
|  | ∆Sleep  Minutes | | ∆Sleep  Efficiency | | ∆Long-Wake  Episodes | | ∆Sleep  Activity | |
|  | *ß* | *SE* | *ß* | *SE* | *ß* | *SE* | *ß* | *SE* |
| Study 1 |  |  |  |  |  |  |  |  |
| Proportional Change | –.50^*^^**^ | .09 | –.71^***^ | .06 | –.72^***^ | .06 | –.60^***^ | .06 |
| Sex | –.16^†^ | .09 | –.08 | .07 | .11 | .07 | –.02 | .08 |
| Body Mass Index | –.10 | .09 | –.10 | .10 | .13 | .10 | .04 | .07 |
| ∆Perceived Economic Wellbeing | .12 | .11 | .15^**^ | .05 | –.19^***^ | .06 | –.23^**^ | .08 |
|  |  |  |  |  |  |  |  |  |
| Fit Indices |  |  |  |  |  |  |  |  |
| χ^2^ | 3.88 |  | 4.32 |  | 4.30 |  | 4.74 |  |
| *df* | 4 |  | 4 |  | 4 |  | 4 |  |
| χ^2^ /*df* | .97 |  | 1.08 |  | 1.08 |  | 1.19 |  |
| RMSEA | .00 |  | .03 |  | .02 |  | .04 |  |
| CFI | 1.00 |  | .99 |  | .99 |  | .99 |  |
|  |  |  |  |  |  |  |  |  |
| Study 2 |  |  |  |  |  |  |  |  |
| Proportional Change | –.64^***^ | .10 | –.34 | .22 | –.27 | .22 | –.24^†^ | .14 |
| Sex | –.01 | .12 | –.01 | .12 | –.05 | .13 | .02 | .13 |
| Body Mass Index | –.14 | .10 | –.06 | .15 | .07 | .19 | –.06 | .17 |
| ∆Perceived Economic Wellbeing | –.07 | .10 | .03 | .13 | –.07 | .12 | –.09 | .12 |
|  |  |  |  |  |  |  |  |  |
| Fit Indices |  |  |  |  |  |  |  |  |
| χ^2^ | 2.81 |  | 1.64 |  | 1.22 |  | 1.63 |  |
| *df* | 4 |  | 4 |  | 4 |  | 4 |  |
| χ^2^ /*df* | .70 |  | .41 |  | .31 |  | .41 |  |
| RMSEA | .00 |  | .00 |  | .00 |  | .00 |  |
| CFI | 1.00 |  | 1.00 |  | 1.00 |  | 1.00 |  |
| *Note*. Study 1: Auburn University Sleep Study. Study 2: Family Stress and Youth Development Study. RMSEA = root mean square error of approximation; CFI = comparative fit index.  ^†^ = *p* < .10. ^*^ = *p* < .05. ^**^ = *p* < .01. ^***^ = *p* < .001. | | | | | | | | |
