# Supplementary material for: From Counting Dollars to Counting Sheep: Exploring Simultaneous Change in Economic Well-Being and Sleep among African American Adolescents
Source: J Racial Ethn Health Disparities. 2024 Oct 22;12(6):4199–208. doi: 10.1007/s40615-024-02212-9 (PMC12644149; doi:10.1007/s40615-024-02212-9)
Supplement: Supplementary file 5 — Supplementary Material 5 [file 40615_2024_2212_MOESM5_ESM.docx]

| *Latent Difference Score Analyses Examining Sleep Outcomes Simultaneously* | | | | | | | | |
| --- | --- | --- | --- | --- | --- | --- | --- | --- |
|  | ∆Sleep  Minutes | | ∆Sleep  Efficiency | | ∆Long-Wake  Episodes | | ∆Sleep  Activity | |
|  | *ß* | *SE* | *ß* | *SE* | *ß* | *SE* | *ß* | *SE* |
| Proportional Change | –.57^***^ | .06 | –.54^***^ | .08 | –.53^***^ | .08 | –.49^***^ | .06 |
| Sex | –.11 | .07 | –.05 | .07 | .04 | .07 | –.01 | .07 |
| Body Mass Index | –.11^†^ | .06 | –.06 | .09 | .07 | .09 | .00 | .07 |
| Study | –.05 | .07 | –.14^*^ | .07 | .12^†^ | .08 | –.05 | .07 |
| ∆Perceived Economic Wellbeing | .06 | .08 | .13^†^ | .07 | –.17^*^ | .07 | –.19^**^ | .08 |
| *Note*. The model was fully saturated. ^†^ = *p* < .10. ^*^ = *p* < .05. ^**^ = *p* ≤ .01. ^***^ = *p* < .001. | | | | | | | | |
