# Supplementary material for: From Counting Dollars to Counting Sheep: Exploring Simultaneous Change in Economic Well-Being and Sleep among African American Adolescents
Source: J Racial Ethn Health Disparities. 2024 Oct 22;12(6):4199–208. doi: 10.1007/s40615-024-02212-9 (PMC12644149; doi:10.1007/s40615-024-02212-9)
Supplement: Supplementary file 6 — Supplementary Material 6 [file 40615_2024_2212_MOESM6_ESM.docx]

| *Latent Difference Score Analyses Examining Sleep Outcomes Individually While Controlling for Time 1 Income-to-Needs* | | | | | | | | |
| --- | --- | --- | --- | --- | --- | --- | --- | --- |
|  | ∆Sleep  Minutes | | ∆Sleep  Efficiency | | ∆Long-Wake  Episodes | | ∆Sleep  Activity | |
|  | *ß* | *SE* | *ß* | *SE* | *ß* | *SE* | *ß* | *SE* |
| Proportional Change | –.56^***^ | .07 | –.59^***^ | .08 | –.54^***^ | .10 | –.50^***^ | .06 |
| Sex | –.10 | .07 | –.06 | .07 | .03 | .07 | –.01 | .07 |
| Body Mass Index | –.10 | .07 | –.05 | .08 | .06 | .09 | –.01 | .07 |
| Study | –.05 | .07 | –.15^*^ | .07 | .13^†^ | .08 | –.05 | .07 |
| Income-to-Needs | .02 | .07 | .07 | .06 | –.07 | .06 | .00 | .06 |
| ∆Perceived Economic Wellbeing | .07 | .08 | .11^†^ | .07 | –.16^*^ | .07 | –.18^*^ | .07 |
|  |  |  |  |  |  |  |  |  |
| Fit Indices |  |  |  |  |  |  |  |  |
| χ^2^ | 17.14^**^ |  | 13.83^*^ |  | 13.70^*^ |  | 14.48^*^ |  |
| *df* | 6 |  | 6 |  | 6 |  | 6 |  |
| χ^2^ /*df* | 2.86 |  | 2.31 |  | 2.28 |  | 2.41 |  |
| RMSEA | .09 |  | .08 |  | .08 |  | .08 |  |
| CFI | .89 |  | .91 |  | .91 |  | .91 |  |
| *Note*. RMSEA = root mean square error of approximation; CFI = comparative fit index. ^†^ = *p* < .10. ^*^ = *p* < .05. ^**^ = *p* < .01. ^***^ = *p* < .001. | | | | | | | | |
